# Phenylacetaldehyde induced olfactory conditioning in *Drosophila melanogaster* (Diptera: Drosophilidae) larvae

**DOI:** 10.1093/jisesa/iead112

**Published:** 2023-12-13

**Authors:** Md Zeeshan Ali, Anushree Anushree, Anwar L Bilgrami, Aarif Ahsan, Mohammad Shamsul Ola, Rizwanul Haque, Jawaid Ahsan

**Affiliations:** Department of Biotechnology, Central University of South Bihar, Gaya, Bihar, India; Department of Biotechnology, Central University of South Bihar, Gaya, Bihar, India; Deanship of Scientific Research, King Abdulaziz University, Jeddah, Saudi Arabia; Department of Radiation Oncology, University of Michigan, Ann Arbor, MI, USA; Department of Biochemistry, College of Science, King Saud University, Riyadh, Saudi Arabia; Department of Biotechnology, Central University of South Bihar, Gaya, Bihar, India; Department of Biotechnology, Central University of South Bihar, Gaya, Bihar, India

**Keywords:** Drosophila melanogaster, ethyl acetate, larvae, learning and memory, phenylacetaldehyde

## Abstract

Phenylacetaldehyde (PAH), an aromatic odorant, exists in varied fruits including overripe bananas and prickly pear cactus, the 2 major host fruits of *Drosophila melanogaster*. It acts as a potent ligand for the Ionotropic receptor 84a (IR84a) and the Odorant receptor 67a (OR67a), serving as an important food and courtship cue for adult fruit flies. *Drosophila melanogaster* larvae respond robustly to diverse feeding odorants, such as ethyl acetate (EA), an aliphatic ester. Since the chemical identity and concentration of an odorant are vital neural information handled by the olfactory system, we studied how larvae respond to PAH, an aromatic food odorant with aphrodisiac properties for adult flies. Our findings revealed that PAH attracted larvae significantly in a dose-dependent manner. Larvae could also be trained with PAH associated to appetitive and aversive reinforcers. Thus, like EA, PAH might serve as an important odorant cue for larvae, aiding in food tracking and survival in the wild. Since IR84a/IR8a complex primarily governs PAH response in adult flies, we examined expression of *Ir84a* and *Ir8a* in early third-instar larvae. Our experiments showed the presence of *Ir8a*, a novel finding. However, contrary to adult flies, PAH-responsive *Ir84a* was not found. Our behavioral experiments with *Ir8a*^*1*^ mutant larvae exhibited normal chemotaxis to PAH, whereas *Orco*^*1*^ mutant showed markedly reduced chemotaxis, indicating an OR-mediated neural circuitry for sensing of PAH in larvae. The results obtained through this study are significantly important as information on how larvae perceive and process PAH odorant at the neuronal level is lacking.

## Graphical Abstract

**Graphical Abstract:**
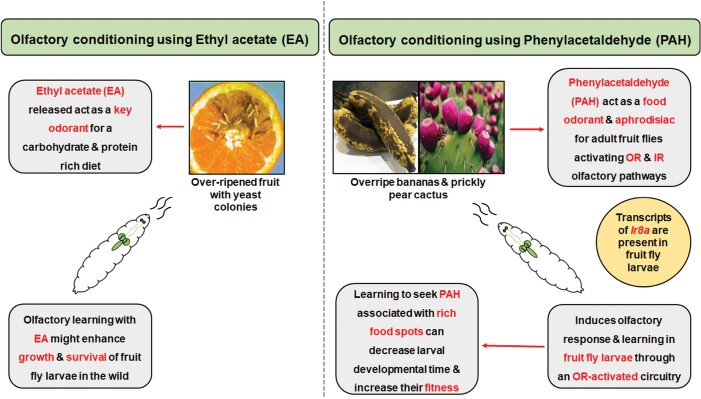

## Introduction

Associative learning and memory formation are critical to the survival of animals in the wild. This capability of the brain allows them to predict important stimuli such as locating food, avoiding predators, seeking suitable partners, and anticipating changes in environmental conditions with past experiences (Hicklen et al. 2010, Thane et al. 2019). Such predictions enable animals to change their behavior to elicit a learned response of vital significance (Scherer et al. 2003, Widmann et al. 2016). For analyzing associative learning and cognition, olfactory classical conditioning has been extensively used by the scientific community using *D. melanogaster* larvae (Aceves-Pina and Quinn 1979, Tully et al. 1994, Honjo and Furukubo-Tokunaga 2005, Khurana et al. 2009, Lesar et al. 2021). Owing to its uncomplicated neuronal architecture, alongside a large array of robust learning paradigms and vast genetic toolkits, the *D. melanogaster* larva is an ideal candidate for deciphering associative plasticity circuitry, physiology, and signaling (Duffy 2002, Ramaekers et al. 2005, Widmann et al. 2016, Komarov and Sprecher 2022).

Phenylacetaldehyde (PAH) is an aromatic compound found in various fruits and other plant tissues including overripe bananas and prickly pear cactus *Opuntia ficus-indica*. These 2 fruits are a vital source of food and oviposition sites for the fly (Grosjean et al. 2011, Ziegler et al. 2013, Sato and Yamamoto 2020). The electrophysiological analyses have revealed that PAH acts as a potent ligand for the Ionotropic receptor 84a (IR84a) and the Odorant receptor 67a (OR67a) in the adult fruit fly (Hallem and Carlson 2006, Benton et al. 2009, Abuin et al. 2011, Silbering et al. 2011). Also, the role of PAH in male courtship behavior through IR84a reiterates its functioning as an environmental aphrodisiac (Grosjean et al. 2011). Thus, PAH, with its ability to activate OR and IR pathways for mediating food and courtship behavior, is a crucial olfactory cue for adult *D. melanogaster* in the wild.

Olfaction in *D. melanogaster* larvae is required for effective foraging and survival to adulthood. They perceive smell through the olfactory system localized to their head region. It consists of a pair of dorsal organs having 21 olfactory sensory neurons (OSNs) each, expressing ORs (Fishilevich and Vosshall 2005, Bose et al. 2015, Ali et al. 2022). Traditionally, olfactory learning paradigms using larvae have employed odorants such as ethyl acetate, 1-butanol, isoamyl acetate, 1-octanol, etc. categorized as aliphatic esters and alcohols (Aceves-Pina and Quinn 1979, Heisenberg et al. 1985, Honjo and Furukubo-Tokunaga 2005, Khurana et al. 2009, Brunner et al. 2020, Thoener et al. 2021). However, studies on dose-dependent olfactory behavior, learning, and cognition of larvae using PAH, an aromatic compound still need to be completed.

As stated, the odorant PAH functions both as a food odorant and an aphrodisiac for adult fruit flies. Since larvae respond well to different food odorants (Heimbeck et al. 1999, Khurana and Siddiqi 2013, Kepchia et al. 2017), it would be interesting to study the olfactory response of larvae to PAH, that unlike normal food odorants has an aphrodisiacal effect on adult flies. This will help identify its functioning as an important odorant stimulus for the larvae too. Also, employing aromatics such as PAH for associative conditioning might help increase the repertoire of odorants that can be used to investigate olfactory learning and memory formation. Therefore, it is of great significance to identify how larvae detect and process PAH at the neuronal level to understand the relevance and perception of aromatic compounds by *D. melanogaster* larvae through behavioral and molecular analyses.

## Materials and Methods

### Fly Stocks

Fly stocks used were the wild-type strain Oregon-R, *Ir8a*^*1*^ mutant (Bloomington *Drosophila* stock number 41744), and *Orco*^*1*^ mutant (Bloomington *Drosophila* stock number 23129), maintained at 25 °C under a day/night cycle of 12 h on standard corn meal media (Lewis 1960). The media comprised of 8 g/l agar (Himedia; 9002-18-0), 15 g/l yeast extract, 80 g/l corn, 20 g/l dextrose (Himedia; 50-99-7), and 40 g/l sucrose (Himedia; 57-50-1). Propionic acid, 4 ml/l (Himedia; 79-09-4), and ortho-phosphoric acid, 0.6 ml/l (Himedia; 7664-38-2), were added as fungicides.

### Chemicals and Reinforcers

The highest grades of chemicals were obtained from Himedia, ThermoFisher, and G-Biosciences. The odorants ethyl acetate (EA) (141-78-6) and phenylacetaldehyde (PAH) (122-78-1) were obtained from Sigma–Aldrich.

### Olfaction, Learning, and Memory Experiments

#### Temperature.

The behavioral experiments on early third-instar larvae were conducted at 25 °C.

#### Isolation of larvae.

**Fig. 1.**
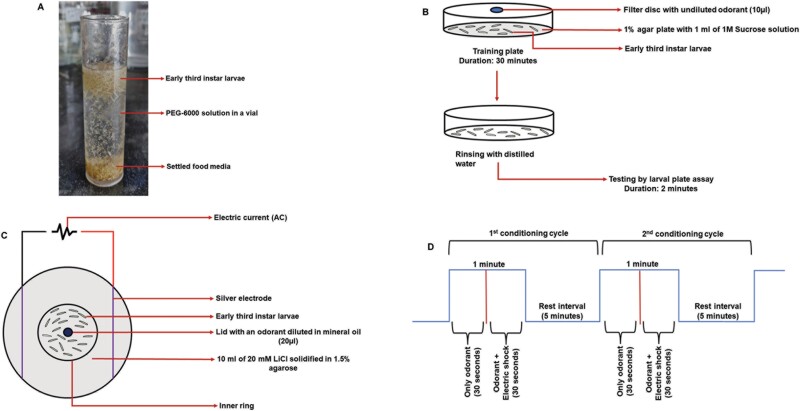
A) Separation of larvae: Larvae along with corn media were put in 30% PEG-6000 solution. Larvae were collected from the top after settling of the food media at the bottom. B) Appetitive olfactory conditioning: ⁓200 early third-instar larvae were put on 1% agar plate with DW (control) or 1 ml of 1M SUC solution (appetitive US). Simultaneously, they were exposed to 10 µl of undiluted odorant (CS) placed on a filter disc inside of the petri plate lid for 30 min. C) Electroshock conditioning: ⁓200 early third-instar larvae were put on a training plate having 10 ml of 20 mM LiCl solidified in 1.5% agarose. The odorant was delivered for 30 s, followed by simultaneous delivery of the odorant and electric shock for another 30 s. A 5-minute rest interval was given. Ten such cycles were run. D) Schematic diagram of the pattern of electroshock conditioning cycles.

Larvae were grown in glass bottles with standard food media as described above. About 150–200 flies were collected in media bottles and left for 20 h for egg-laying. All the experiments were conducted using early third-instar larvae (72 h after egg-laying) to ensure a uniform population. Larvae were harvested by scraping the top layer of the food media in water with a paintbrush and collected on a sieve (aperture, 500 μm) to remove the fine debris. The larvae along with the coarse media were then transferred to a vial holding 30% polyethylene glycol-6000 (PEG-6000) (Himedia; 25322-68-3) solution for density separation. Doing so, the larvae swam to the top, while the corn settled down at the bottom (Khurana 2003, Khurana et al. 2009) (Fig. 1A). The top layer was transferred to the sieve and rinsed twice with distilled water (DW) to remove any PEG-6000 stuck to the larval bodies. Finally, the larvae were transferred to a Petri plate (Borosil) holding 0.5 ml of Ringer’s solution to prevent desiccation until the experiment began. The Ringer’s solution was made up of 128 mM NaCl (Himedia; 7647-14-5), 4.7 mM KCl (Himedia; 7447-40-7), 1.8 mM CaCl_2_ (Himedia; 10035-04-8), 0.9 mM Na_2_HPO_4_ (Himedia; 7558-79-4), and 0.37 mM KH_2_PO_4_ (Himedia; 7778-77-0) (Robb 1969, Khurana et al. 2010).

#### Appetitive olfactory conditioning.

Appetitive olfactory conditioning was done similarly to Honjo and Furukubo-Tokunaga (2005). About 1 ml of DW (control) or 1 M sucrose (SUC) solution (training) was spread on 1% of newly prepared agar plates (90 mm glass Petri dishes). The harvested larvae (approximately 200 in number) were transferred from the Ringer’s solution to the training plate with the help of a paintbrush. A filter disc (15 mm diameter) prepared by cutting a filter paper (Whatman; 1001-125) was placed inside the lid with 10 µl of neat odorant on it. The plate was immediately covered with the lid, and the setup was left undisturbed for 30 min. This resulted in simultaneous exposure of larvae to both the odorant (CS) and the sucrose (US) (Fig. 1B). After 30 min, the larvae were transferred to a plate holding DW and rinsed properly to remove any residual sucrose or odorant adhering to their bodies. Lastly, the larvae were transferred to the testing plates. Although the mineral oil used for diluting the odorants was of the highest grade (odorless and tasteless), a vehicle control (associating mineral oil with sucrose) was performed to neutralize any influence of mineral oil on the larval behavior. Larvae naive to CS were used as an additional control.

#### Aversive olfactory conditioning (electroshock conditioning).

Electroshock conditioning was performed as per Khurana et al. (2009). About 1.5% agarose (Himedia; 9012-36-6) was dissolved in 20 mM lithium chloride (LiCl) (Himedia; 7447-41-8) solution by heating in a conical flask. About 10 ml of the molten agarose was poured into a 9 cm Petri dish (training plate) fitted with 2 diametrically opposite silver electrodes. Since the LiCl solution is a good conductor of electricity, the current passing through the electrodes could run across the agarose surface. At partial solidification, a ring made by removing the base of a 4.5-cm Petri dish was placed in the middle to make a confinement zone. Approximately 200 early third-instar larvae were placed in the confinement zone touching the agarose surface and bathed with 500 µl of 20 mM LiCl solution to ensure that the larvae experience the required electric shock. For odorant delivery, 20 µl of an appropriately diluted odorant (CS) in mineral oil was placed inside a lid and put on the inner ring (Fig. 1C). The odorant was delivered for 30 s, followed by 30 s of simultaneous exposure to odorant and the alternating current (AC) as electric shock. About 110 V of AC was applied through the electrodes using a voltage converter. The voltage gradient (14 V/cm) was measured using a multimeter by placing it at different areas on the training plate. At the end of 1 min, the lid with the odorant was removed and replaced with a blank lid. A 5-minute rest interval was given to the larvae during which they experienced neither odorant nor shock. This completed a single trial. Ten such trials were done (Fig. 1D). Finally, the larvae were transferred to the testing plates. Three types of controls were used in this experiment; only odorant (larvae exposed to only odorant at room temperature), only electric shock (larvae subjected to only electric shock devoid of any odorant stimulation), and a vehicle control (associating mineral oil with electric shock) to neutralize any influence of mineral oil on the larval behavior.

### Quantification of Larval Response, Learning, and Memory

**Fig. 2.**
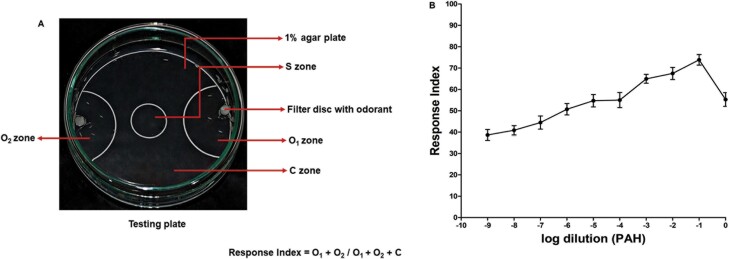
A) Testing: Nearly 50 larvae were put in the S zone. About 20 µl of an odorant diluted to the appropriate concentration in mineral oil was placed on the filter discs in the O_1_ and O_2_ zones each. After 2 min, the number of larvae in various demarcated zones were counted, and RI was calculated. B) Larval response to various dilutions of PAH: Attraction reached the largest at 10^−1^. *N* = 10 for each dilution point. Error bars presented are the standard deviation of the mean (SDM).


The quantification of olfactory response and measurement of learning and memory acquired by larvae after conditioning was done using the larval plate assay adapted from Khurana et al. (2009). Fifty larvae were put in the middle of a Petri dish (9 cm) containing 10 ml of Ringer’s solution solidified in 1% agar. About 20 µl of the odorant diluted in mineral oil (Himedia; 8042-47-5) was placed on each paper disc (5 mm diameter), placed diametrically opposite in the 2 arcs of radius 2 cm from the plate’s margin (Fig. 2A). At the end of 2 min, pictures were taken to decipher the larval counts in the various demarcated zones for calculating the response and the learning indices.

$$\begin{array}{l} Response\;index\left(RI\right):\;\frac{\begin{array}{l} Number\;of\;larvae\;in\;zone\;1\;\left(O_{1}\right)+\;Number\;of\;larvae\;in\;zone\;2\;\left(O_{2}\right) \end{array}}{Total\;number\;of\;larvae\;\left(O_{1}+\;O_{2}+\;C\right)} \end{array}$$

$$\text{Learning index }\left(\text{LI}\right)\left(\text{Appetitive learning}\right):\;\frac{{\text{Response index}}_{\text{Trained}}-{\text{Response index}}_{\text{Control}}}{{\text{Response index}}_{\text{Control}}}$$

$$\text{Learning index }\left(\text{LI}\right)\left(\text{Aversive learning}\right):\;\frac{{\text{Response index}}_{\text{Control}}-{\text{Response index}}_{\text{Trained}}}{{\text{Response index}}_{\text{Control}}}$$

### Statistics

The significance of differences between conditioned and unconditioned responses was estimated by parametric tests (Student’s *t-*test and ANOVA). Before these parametric tests, normality of the data sets was tested with the help of Shapiro–Wilk test (<50 samples). In case of non-normal distribution, transformation of the data sets was performed using GraphPad Prism. The data were also cross-checked by nonparametric tests (Mann–Whitney *U* test or the Kruskal–Wallis test) owing to the small sample size to confirm the statistical significance further. The conclusions were unchanged between the parametric and nonparametric tests. Error bars presented in the entire paper are the standard deviation of the mean (SDM).

### Total RNA Extraction from Larvae

Total RNA was isolated from early third-instar larvae using a protocol similar to Bogart and Andrews (2006), with slight modifications. About 50 mg of frozen early third-instar larvae were taken in a 1.5 ml Eppendorf tube with 1 ml Trizol reagent (ThermoFisher; 15596026). It was at once homogenized with a plastic pestle. After incubating at room temperature for 5 min, the tube was centrifuged at 12,000 rcf for 10 min at 4 °C to settle insoluble debris. The supernatant was transferred to a fresh tube carrying 200 µl of chloroform (Himedia; 67-66-3). After shaking vigorously by hand, the tube was incubated at room temperature for 3 min and centrifuged again at 10,000 rcf for 15 min at 4 °C. The upper layered aqueous phase was transferred to a fresh tube. About 500 µl of isopropanol (Himedia; 67-63-0) was added to it followed by a 10-min incubation at room temperature. The tube was then centrifuged at 12,000 rcf for 10 min at 4 °C. After removing the supernatant, the pellet was washed with 1 ml of 75% ethanol (Himedia; 64-17-5). The tube was centrifuged again at 7,500 rcf for 5 min at 4 °C, and the supernatant was discarded. This was followed by a brief centrifugation of the tube to remove the last of the supernatant using a micropipette. It was then left for 10 min to be air-dried. At last, the pellet was dissolved in 100 µl of molecular grade water (Himedia).

### Reverse Transcription

Larval cDNA was prepared from the total RNA using a HiGenoMB cDNA synthesis kit (Himedia) as per the manufacturer’s directives.

### Standard Polymerase Chain Reaction

For standard polymerase chain reaction (PCR), 0.5 µM final concentrations of both forward and reverse primers (1.25 µl each) (G-Biosciences), 1× final concentration of PCR buffer (2.5 µl) (G-Biosciences; 786-447), 0.4 mM final concentration of dNTP mix (1 µl) (G-Biosciences; 786-443), 2.5 U of Taq DNA Polymerase (0.5 µl) (G-Biosciences; 786-447), molecular grade water (16.5 µl) (Himedia), and cDNA (25 ng/µl) (2 µl) per 25 µl of total reaction volume were used. PCR was performed on a ProFlex PCR system (Applied Biosystems) as follows: 95 °C for 7 min; 35 cycles of 95 °C for 1 min, 55 °C for 30 s, and 72 °C for 30 s; and a final extension step at 72 °C for 10 min. The products were run on 2% ultrapure agarose gel (Himedia; 9012-36-6) in 1× Tris-acetate-EDTA buffer with DNAmark 100bp Plus Ladder (G-Biosciences; 786-856). PCR was performed in 3 technical replicates for each cDNA sample.

The following intron spanning primers, designed by Oligo Explorer 1.2 software, were used besides *dRP49* (reference gene):

*Ir84a*: Forward Primer—5ʹ-TGCACACCCAGAACATCCAC-3ʹ
  Reverse Primer—5ʹ-GACCAAAGCCAGGACACACC-3ʹ
*Ir8a*: Forward Primer—5ʹ-GATAACCTTTTGGATTsGAGCC-3ʹ
  Reverse Primer—5ʹ-CTTAACATCCAGACGCAGTG-3ʹ
*dRP49*: Forward Primer—5ʹ-TCTGATGCCCAACATCGGTT-3ʹ
  Reverse Primer—5ʹ-TCTCCTTGCGCTTCTTGGAG-3ʹ

### Quantitative Polymerase Chain Reaction

For quantitative polymerase chain reaction (qPCR), a 20 µl total PCR reaction volume was prepared using 0.5 µM final concentrations of both forward and reverse primers (1 µl each) (G-Biosciences), Hi-SYBr Master Mix (10 µl) (Himedia), molecular grade water (6 µl), and cDNA (25 ng/µl) (2 µl). Real-time qPCR was done using a Rotor-Gene Q Real-Time PCR Detection System (Qiagen) as follows: 95 °C for 10 min; 40 cycles of 95 °C for 30 s, 60 °C for 35 s, and 72 °C for 40 s; and a final extension step at 72 °C for 5 min. The *C*_*t*_ values were recorded with Rotor-Gene Q Software 2.3.5.1. A melt curve analysis was also done after the assay to check for the specificity of the reaction. qPCR was performed in 3 technical replicates for each cDNA sample. The gel electrophoresis of qPCR products was also done.

## Results

### Dose–Response Curve of PAH

The complete olfactory behavioral dose–response curve of PAH for early third-instar larvae was prepared using the larval plate assay as described in materials and methods. The larval response reached a maximum at 10^−1^ dilution (Fig. 2B). The dilution 10^−2^ was chosen for learning and memory experiments with PAH.

### Appetitive Olfactory Conditioning With Single Odorants

**Fig. 3.**
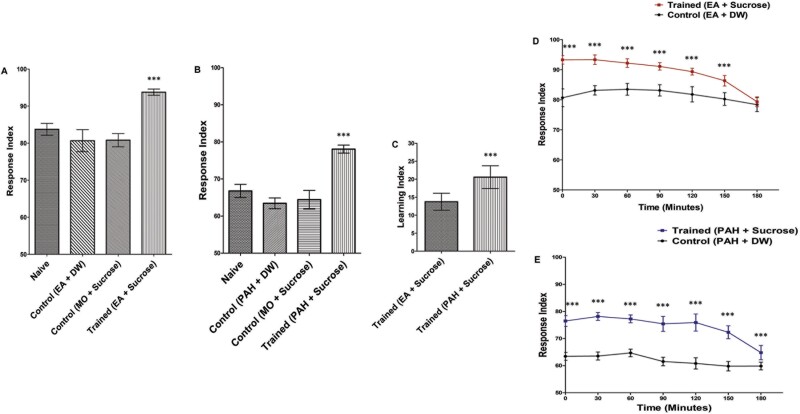
Characterization of appetitive conditioning in *D. melanogaster* larvae: A significant increment in RI was seen when A) EA was associated with 1 M sucrose and B) PAH was associated with 1 M sucrose. ***P < 0.0001 with ANOVA compared to any of the 3 other conditions; also cross-checked by the Kruskal–Wallis test, *P* < 0.0001. C) Learning acquired by PAH/SUC was significantly greater than EA/SUC. ***P < 0.0001 with Student’s *t*-test, also cross-checked by the Mann–Whitney *U* test, *P* < 0.001. D) Memory retention curve of appetitive conditioning with EA/SUC and control conditioning with EA/DW. ***P < 0.0001 with Student’s *t*-test, also cross-checked by the Mann–Whitney *U* test, *P* < 0.05. E) Memory retention curve of appetitive conditioning with PAH/SUC and control conditioning with PAH/DW. ***P < 0.0001 with Student’s *t*-test, also cross-checked by the Mann–Whitney *U* test, *P* < 0.001. *N* = 10 for each experiment. Error bars presented are the standard deviation of the mean (SDM).

As described in the methods section, early third-instar larvae were conditioned to associate EA and PAH with 1 M sucrose. After training, the larvae were tested at specific time intervals using the larval plate assay to decipher learning and memory acquisition. Three different controls were used (a) naive, that is, larvae exposed neither to an odorant nor sucrose, (b) larvae trained to associate odorants with DW, that is, ethyl acetate + distilled water (EA/DW) and phenylacetaldehyde + distilled water (PAH/DW), and (c) a vehicle control, that is, associating mineral oil with sucrose. The association of the odorants with sucrose, that is, ethyl acetate + sucrose (EA/SUC) and phenylacetaldehyde + sucrose (PAH/SUC) led to a significant increment in the RI of the trained larvae (Fig. 3A and B). The LI was also calculated (Fig. 3C). The learning acquired by the association of PAH/SUC was significantly greater as compared to EA/SUC.

### Memory Retention Curve of Appetitive Conditioning

The stability of the larval memory formed after appetitive conditioning was analyzed by studying the memory decay curve for up to 3 h. The response indices of the larvae were measured at once after training and then at 30-minute intervals for up to 180 min. The association of EA and PAH with 1M sucrose (EA/SUC; PAH/SUC) both resulted in an increment of the response indices of the larvae. This increase compared to the controls (EA/DW; PAH/DW) exhibited a significant memory formation. The memory formed showed a gradual decay and was completely lost by 180 min in case of EA/SUC whereas, it remained significant even 180 min after conditioning in case of PAH/SUC (Fig. 3D and E).

### Aversive Olfactory Conditioning with Single Odorants

**Fig. 4.**
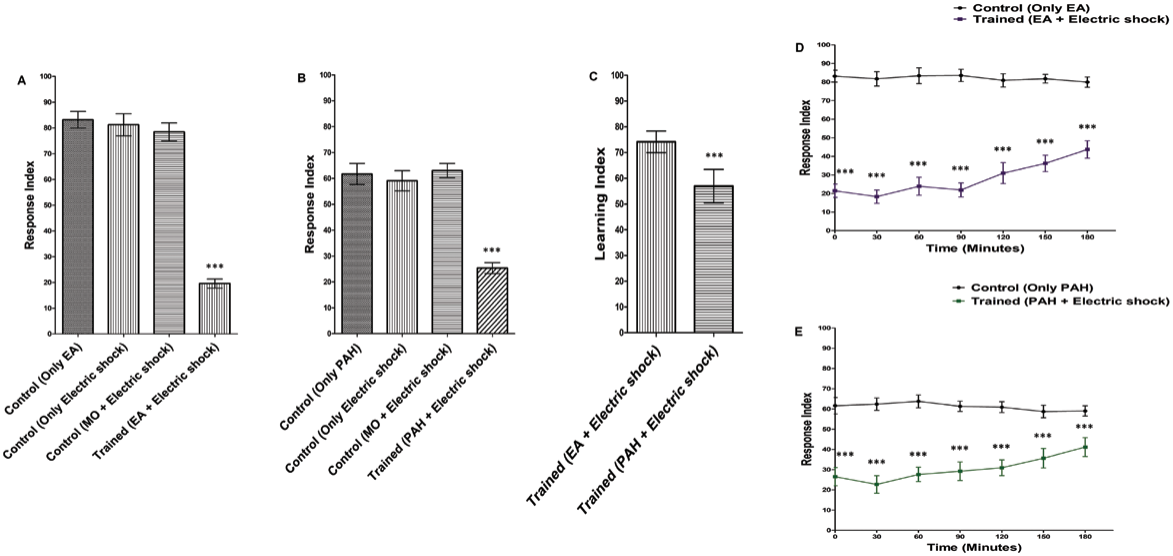
Characterization of aversive conditioning in *D. melanogaster* larvae: A significant decrement in RI was seen when A) EA was associated with electric shock and B) PAH was associated with electric shock. ***P < 0.0001 with ANOVA compared with any of the 3 other conditions; also cross-checked by the Kruskal–Wallis test, *P* < 0.0001. C) Learning acquired by EA/Electric shock was significantly greater than PAH/Electric shock. ***P < 0.0001 with Student’s *t*-test; also cross-checked by the Mann–Whitney *U* test, *P* < 0.0001. D) Memory retention curve of aversive conditioning with EA/Electric shock and control conditioning with only EA. E) Memory retention curve of aversive conditioning with PAH/Electric shock and control conditioning with only PAH. ***P < 0.0001 with Student’s *t*-test, also confirmed by the Mann–Whitney *U* test, *P* < 0.001. *N* = 10 for each experiment. Error bars presented are the standard deviation of the mean (SDM).

The aversive olfactory conditioning was performed by associating EA and PAH with electric shock. After training, the larvae were tested at specific time intervals using the larval plate assay to decipher learning and memory acquisition. Three different controls were used (i) larvae exposed only to an odorant, (ii) larvae exposed only to electric shock, and (iii) a vehicle control (associating mineral oil with electric shock). The association of the odorants with electric shock (EA + Electric shock; PAH + Electric shock) led to a significant decrement in the RI of the trained larvae (Fig. 4A and B). The LI was also calculated (Fig. 4C). The learning acquired by the association of EA + Electric shock was significantly greater than PAH + Electric shock.

### Memory Retention Curve of Aversive Conditioning

The stability of the larval memory formed after aversive conditioning was analyzed by studying the memory decay curve for up to 3 h. The response indices of the larvae were measured at once after training and then at 30-minute intervals for up to 180 min. The association of EA with electric shock resulted in a decrement in the RI of the trained larvae. This decrease compared to the controls exhibited a significant formation of memory. The memory formed displayed a gradual decay though remained significant even 180 min after conditioning (Fig. 4D). The association of PAH with electric shock also showed a similar dip in the RI and pattern of memory decay (Fig. 4E).

### *Ir84a* and *Ir8a* Expression in *D. melanogaster* Larvae

**Fig. 5.**
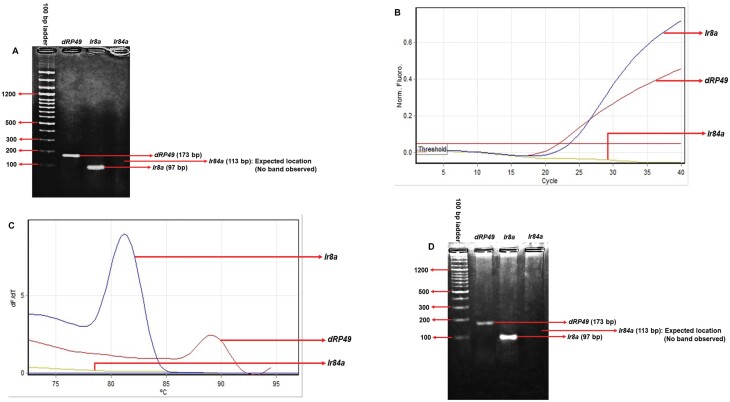
A) Gel photograph showing different sizes (in base pairs; bp) and intensities of standard PCR products of *Ir8a* and *dRP49*, run with a 100 bp ladder. B) Curves showing amplification of *Ir8a* and *dRP49* (in terms of normalized fluorescence) as a function of the number of cycles. C) Melt curves of qPCR products of *Ir8a* and *dRP49*. Different peaks specify different melting temperatures of different PCR products. D) Gel photograph showing different sizes (in base pairs; bp) and intensities of qPCR products of *Ir8a* and *dRP49*, run with a 100 bp ladder. RNA was extracted from early third-instar larvae. *N* = 6 for each PCR run.

In adult *D. melanogaster*, the olfactory response to PAH is primarily mediated by the IR84a/IR8a receptor complex (Benton et al. 2009, Abuin et al. 2011). So, the expression of *Ir84a and Ir8a* besides *dRP49* (reference gene) was checked in the early third-instar larva by the traditional standard PCR method. The traditional PCR produced distinct bands for *Ir8a* and *dRP49* (Fig. 5A). To further confirm the results, a much more sensitive qPCR was employed. In qPCR, a C_t_ value of 23.59 and 22.02 for *Ir8a* and *dRP49* respectively was recorded (Fig. 5B). Besides, the melt curve analysis gave peaks at 81.2 °C and 89.0 °C for *Ir8a* and *dRP49* respectively (Fig. 5C). Gel electrophoresis of the qPCR products producing distinct bands for the 2 genes was also performed (Fig. 5D). However, in both traditional PCR and qPCR no expression of *Ir84a* was detected.

### Olfactory Response of the *Ir8a*^*1*^ Mutant Larvae to PAH

**Fig. 6.**
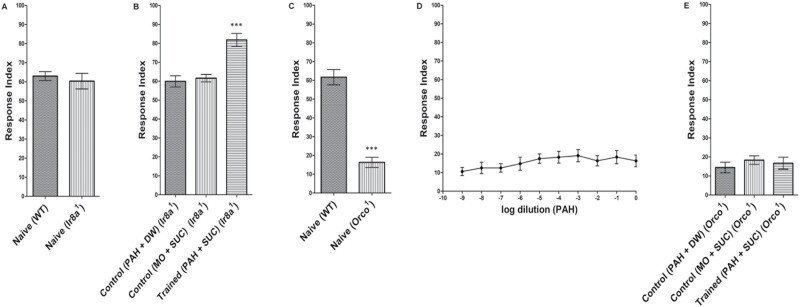
A) Olfactory response of the *Ir8a*^*1*^ mutant larvae to PAH (10^−2^) in contrast to the WT larvae. The *Ir8a*^*1*^ mutant exhibited normal chemotaxis to PAH. B) Appetitive olfactory classical conditioning with PAH/SUC using *Ir8a*^*1*^ mutant larvae. A significant learning was acquired by them. ***P < 0.0001 with ANOVA compared with any of the 2 other conditions, also cross-checked by the Kruskal–Wallis test, *P* < 0.0001. C) Olfactory response of the *Orco*^*1*^ mutant larvae to PAH (10^−2^) in comparison to the WT larvae. The *Orco*^*1*^ mutant exhibited remarkably decreased olfactory response towards PAH. ***P < 0.0001 with Student’s *t*-test, also cross-checked by the Mann–Whitney *U* test, *P* < 0.0001. D) *Orco*^*1*^ mutant larval response to different dilutions of PAH. The *Orco*^*1*^ larvae exhibited a highly reduced olfactory response to various dilutions of the PAH used. E) Appetitive olfactory classical conditioning with PAH/SUC using *Orco*^*1*^ mutant larvae. No associative learning was acquired by them. *N* = 10 for each experiment. Error bars presented are the standard deviation of the mean (SDM).

The olfactory response of the *Ir8a*^*1*^ (*Ir8a*−/−) mutant larvae to PAH (10^−2^) was measured by using the larval plate assay. The mutant larvae exhibited normal chemotaxis towards PAH compared to the wild-type (WT) larvae (Fig. 6A). Further, the *Ir8a*^*1*^ mutant larvae were also used to perform appetitive olfactory conditioning with PAH/SUC. A significant learning was acquired by them (Fig. 6B).

### Olfactory Response of the *Orco*^*1*^ Mutant Larvae to PAH

Similarly, the olfactory response of the *Orco*^*1*^ (*Orco*−*/*−) mutant larvae to PAH (10^−2^) was determined using the larval plate assay. The mutant larvae showed a severely reduced chemotaxis towards PAH compared to the wild-type (WT) larvae (Fig. 6C). Further, the complete olfactory behavioral dose-response curve of PAH for the *Orco*^*1*^ larvae was prepared. The larval response was distinctly reduced across different dilutions of PAH (Fig. 6D). The *Orco*^*1*^ mutant larvae were also used to perform appetitive olfactory conditioning with PAH/SUC. No learning was acquired by them (Fig. 6E).

## Discussion

*Drosophila melanogaster* larvae are robustly attracted to food odorants, such as ethyl acetate (EA), an aliphatic ester at right dilutions (Khurana et al. 2009, Khurana and Siddiqi 2013, Kepchia et al. 2017). PAH is an aromatic compound present in 2 significant host fruits for *D. melanogaster*, that is, overripe bananas and prickly pear cactus (Markow and Grady 2008, Grosjean et al. 2011). Because the chemical identity and concentration of an odorant represent significant neural information handled by the olfactory system, we hypothesized whether or not PAH, which, unlike general food odorants, is known to promote male courtship behavior in adult fruit flies, elicits an olfactory response by larvae. Our study proved that the early third-instar larvae were strongly attracted to PAH. The attraction (Response Index) peaked at a dilution of 10^−1^ when aversion sets in. Therefore, the dilution right after the aversion threshold that is, 10^−2^ was chosen in order to study an increase in attraction (appetitive olfactory conditioning) or increase in aversion that is, loss of attraction (aversive olfactory conditioning).

Further, in the present study, we have characterized larval appetitive memory formed by EA and PAH with sucrose as the unconditioned stimulus (US). The study demonstrated that likewise aliphatic ester EA, the association of aromatic compound PAH with sucrose led to a significant increment in the RI of the trained larvae. We further characterized larval aversive memory formed by EA and PAH with electric shock as the aversive stimuli. We found that similar to EA, the association of PAH with electric shock led to a significant decrease in RI of the trained larvae. Both increment and decrement in the RI highlighted the induction of learning and memory consolidation. Thus, the study marked the use of aromatics besides aliphatic esters and alcohols for olfactory associative conditioning. In due course, more work in this direction will help increase the repertoire of odorants that can be used to study olfactory learning and memory formation and also decipher the significance of aromatic compounds for *D. melanogaster* larvae in the wild.

Under natural conditions, *D. melanogaster* feeds on rotten fermenting fruits that serve as sites for their courtship and egg-laying too. These fruits are rich carbohydrate sources critical for the life span, fecundity, and subsistence of fruit flies (Lee 2015). The spoiled fruits also serve as a substrate for yeast, an important source of dietary proteins, especially during the larval stage (Becher et al. 2012, Grangeteau et al. 2018, Lewis and Hamby 2019). Since larvae are deposited on the food source, their growth is presumably possible even without associative learning. However, fruit fly larvae possess the ability to evaluate environmental parameters such as high-quality nutritional sources or predation by learning to associate them with relevant odorant stimuli (Dukas 1999, Das et al. 2016). Subsequently, the capacity of learning to seek an aromatic odorant such as PAH associated with rich food spots can decrease larval developmental time and increase their fitness in the wild.

Besides, the response to PAH in adult *D. melanogaster* is primarily mediated by the IR84a/IR8a heteromeric complex (Benton et al. 2009, Abuin et al. 2011). So, we checked the expression of *Ir84a and Ir8a* in early third-instar larvae owing to their olfactory response and associative learning with PAH. Our results were under the previously published data confirming the nonexistence of IR84a in the larval stage (Sanchez-Alcaniz et al. 2018). However, contrary to the earlier published reports indicating the absence of IR8a in larva with Ir-Gal4 driven mCD8:GFP expression (Stewart et al. 2015, Croset et al. 2016, Sanchez-Alcaniz et al. 2018), we found the expression of *Ir8a* in the larval stage using much more sensitive PCR technique. Both the standard and qPCR data showed the presence of transcripts of *Ir8a* in the *D. melanogaster* larva.

In adult *D. melanogaster*, IR8a is a widely expressed olfactory co-receptor found in the third antennal segment and the third chamber of the sacculus. It forms complex with tuning IRs to detect diverse odorants, as observed in the detection of PAH, where it complexes along IR84a (Abuin et al. 2011, Ni 2021). With IR84a being absent at the larval stage, we checked the participation of IR8a in the sensing of PAH by larvae using the *Ir8a*^*1*^ mutant. The *Ir8a*^*1*^ mutant larvae exhibited normal chemotaxis to PAH comparable to the WT larvae and were able to acquire significant learning with PAH/SUC olfactory conditioning. These results negated any role of IR8a in the larval sensing of PAH.

Since there is no known expression of tuning IRs in larval OSNs other than IR92a, which mediates the olfactory response to ammonia (Rimal and Lee 2018, Ni 2021), the involvement of ORs in larval response to PAH was investigated. For this, we used early third-instar larvae of *Orco*^*1*^ mutant flies. Orco is a broadly expressed olfactory co-receptor that partners with conventional ORs to form a functional receptor complex for ligand binding. It acts as a protein chaperone, guiding ORs to dendrites and serving as a link to the OSN signal transduction cascade. Loss of Orco in *D. melanogaster* causes disrupted localization of OR proteins in chemosensory dendrites in both larval and adult olfactory systems. This results in significant impairment in the detection of a variety of OR-responsive odorants. As a consequence, *Orco*^*1*^ mutant larvae are unable to navigate toward OR-responsive odorants, and adult flies display notable deficiencies in odor-triggered electrophysiology and behavior (Larsson et al. 2004). Subsequently, our results unveiled that the *Orco*^*1*^ mutant larvae exhibited severely reduced chemotaxis to PAH. Moreover, they also failed to acquire any learning with PAH/SUC olfactory conditioning. These results indicate that at the larval stage, olfactory response, and associative learning with PAH is mediated by an OR-dependent olfactory pathway.

Because IRs are multimodal entities mediating olfaction, gustation, thermosensation, and hygrosensation in *D. melanogaster*, a further analysis of the transcripts of *Ir8a*, the gene encoding the IR8a co-receptor might unveil some new dimensions of the larval neural system as the receptor cannot be redundant in function.

## Data Availability

The datasets used and/or analyzed during the current study are available from the corresponding author on reasonable request.
